# St. Nicholas

**Published:** 1875-01

**Authors:** 


*St. Nicholas*

Is the most splendid juvenile magazine in the world. It is highly illustrated, and the reading matter is the purest and best. Every boy and girl should have *St. Nicholas*. Send to Scribner & Co., New York. Price, $5.00.

